# Platelet-derived exosomes from septic shock patients induce myocardial dysfunction

**DOI:** 10.1186/cc6176

**Published:** 2007-11-08

**Authors:** Luciano Cesar Pontes Azevedo, Mariano Janiszewski, Vera Pontieri, Marcelo de Almeida Pedro, Estevão Bassi, Paulo José Ferreira Tucci, Francisco Rafael Martins Laurindo

**Affiliations:** 1 Emergency Medicine Research Laboratory, University of São Paulo School of Medicine, (Av. Dr. Enéas de Carvalho Aguiar 255, sala 5023, ZIP: 05403-900, São Paulo, Brazil); 2 Research and Education Institute, Hospital Sírio-Libanês, (R. Cel Nicolau dos Santos 69, ZIP: 01308060, São Paulo, Brazil); 3 Pharmacology Department, Biomedical Sciences Institute, University of São Paulo, (Av. Prof. Lineu Prestes, 1.524, 2° andar, sala 215 ZIP:05508-900 – São Paulo, Brazil); 4 Vascular Biology Laboratory, Heart Institute – InCor, University of São Paulo School of Medicine, Av. Eneas Carvalho Aguiar, 44-subsolo, ZIP 05403-000 São Paulo, Brazil); 5 Cardiovascular Physiology Laboratory, Federal University of São Paulo, (R. Estado de Israel, 181 ZIP:04022-000 – São Paulo, SP – Brazil)

## Abstract

**Introduction:**

Mechanisms underlying inotropic failure in septic shock are incompletely understood. We previously identified the presence of exosomes in the plasma of septic shock patients. These exosomes are released mainly by platelets, produce superoxide, and induce apoptosis in vascular cells by a redox-dependent pathway. We hypothesized that circulating platelet-derived exosomes could contribute to inotropic dysfunction of sepsis.

**Methods:**

We collected blood samples from 55 patients with septic shock and 12 healthy volunteers for exosome separation. Exosomes from septic patients and healthy individuals were investigated concerning their myocardial depressant effect in isolated heart and papillary muscle preparations.

**Results:**

Exosomes from the plasma of septic patients significantly decreased positive and negative derivatives of left ventricular pressure in isolated rabbit hearts or developed tension and its first positive derivative in papillary muscles. Exosomes from healthy individuals decreased these variables non-significantly. In hearts from rabbits previously exposed to endotoxin, septic exosomes decreased positive and negative derivatives of ventricular pressure. This negative inotropic effect was fully reversible upon withdrawal of exosomes. Nitric oxide (NO) production from exosomes derived from septic shock patients was demonstrated by fluorescence. Also, there was an increase in myocardial nitrate content after exposure to septic exosomes.

**Conclusion:**

Circulating platelet-derived exosomes from septic patients induced myocardial dysfunction in isolated heart and papillary muscle preparations, a phenomenon enhanced by previous *in vivo *exposure to lipopolysaccharide. The generation of NO by septic exosomes and the increased myocardial nitrate content after incubation with exosomes from septic patients suggest an NO-dependent mechanism that may contribute to myocardial dysfunction of sepsis.

## Introduction

Septic shock remains one of the most challenging medical conditions, with increasing incidence over the last years. The tendency of such an increase is probably due to progressive aging of the population, improvements in critical care support, and progress in chemotherapy and immunosuppressive therapies, with increased life expectancy of immunosupressed patients and patients with malignancies. Although much has been learned about the pathophysiology of sepsis in the last decade, the mortality of this condition is still elevated.

One of the most important features of sepsis is myocardial dysfunction [1-3]. Studies with echocardiogram and radionuclides show biventricular dilatation and decrease in ejection fraction in septic patients [3]. This myocardial depression is reversible in most individuals after 7 to 10 days, and the exact correlation of this dysfunction with the prognosis is incompletely understood [4].

Several hypotheses have been formulated to explain the decrease in myocardial function in sepsis. Cytokines such as interleukin (IL)-1 and tumor necrosis factor-alpha (TNF-α) [5] and, more recently, reactive oxygen/nitrogen species (nitric oxide [NO], superoxide, and peroxynitrite) have been implicated in the mechanism [6-8], with studies showing a decrease in myocardial contractility after exposure to such intermediates. However, the search for a specific protein characterized as 'sepsis myocardial depressant factor' continues, with controversial results.

A previous report from our laboratory showed the presence in the plasma of septic patients of microparticles characterized as exosomes [9]. These particles were characterized to be predominantly derived from platelets. Microparticles are vesicular or lamellar structures released by several cell lines after activation or apoptosis, which have been related to vascular dysfunction and activation of coagulation in sepsis [10,11]. Exosomes are a particular type of microparticles produced by the endocytic-lysosomal system of several cell lines after activation [12]. Platelet-derived exosomes are lamellar particles with diameters of approximately 100 nm with few procoagulant properties which do not bind annexin V. The presence of tetraspan proteins (such as CD37 and CD63) in exosome membrane suggests that they may have signaling and adhesion purposes, although the exact function of these particles needs to be determined [9,13]. The previous study showed that exosomes derived from septic shock patients possess NAD(P)H oxidase activity identified by lucigenin-induced chemiluminescence and cytochrome c reduction assay and exhibit a potent proapoptotic effect in endothelial and smooth muscle vascular cells through a redox-dependent pathway [9]. Since redox mechanisms may be involved in cardiovascular dysfunction of sepsis [14], we conducted this study in order to evaluate a possible role for platelet-derived exosomes in sepsis-related myocardial dysfunction and possible redox mechanisms underlying such effects.

## Materials and methods

All chemicals and reagents were purchased from Sigma-Aldrich (St. Louis, MO, USA) unless otherwise specified.

### Preparation of exosomes

We collected blood samples (50 mL) of 55 patients from the Emergency Medicine Intensive Care Unit of Hospital das Clinicas, University of São Paulo, Brazil, with early (less than 48 hours) diagnosis of septic shock defined according to the criteria of the American College of Chest Physicians and the Society of Critical Care Medicine [15]. Twelve healthy volunteers from our laboratory provided blood samples that served as controls. The study was approved by the institutional ethics and review board, and informed consent was obtained from the patient's next of kin. Animal care and handling of animals in the study were in accordance with guidelines of the National Institutes of Health (Bethesda, MD, USA).

The technique for isolating exosomes was performed as described previously [9]. Briefly, blood was collected in centrifuge tubes containing 5 U/mL heparin, and all procedures were carried out at 4°C. Cells, platelets, and large debris were pelleted by centrifugation at 3,000 *g *for 10 minutes. Three (3.0) mM phenylmethylsulfonyl fluoride (PMSF), 1 μg/mL aprotinin, and pepstatin were added to the supernatant, which then was sequentially filtered through 1-, 0.45-, and 0.22-μm nylon filters to remove platelets, cellular fragments, and apoptotic bodies. The remaining cell-free plasma was ultracentrifuged at 140,000 *g *for 150 minutes. The pellet containing exosomes first was washed with Tris (50 mM)-buffered saline (NaCl 150 mM) with ethylenediaminetetraacetic acid (EDTA) (0.1 mM) to avoid contamination with plasma proteins and then was resuspended with the same buffer. Protein concentration was measured by the Bradford method (Bio-Rad Laboratories GmbH, München, Germany). After separation procedures, healthy individuals generated samples with 5.8 ± 0.96 mg of exosome protein per milliliter of solution, whereas septic patients generated samples with 16.5 ± 0.99 mg of exosome protein per milliliter of solution (*P *< 0.05, *t *test).

### Electron microscopy

In experiments involving electron microscopy, exosome separation was performed without the steps of filtration and ultracentrifugation, in order to avoid aggregation of exosomes. Briefly, the plasma of septic patients was centrifuged at 11,000 *g *for 2 minutes to obtain platelet-poor plasma. A buffer containing Tris-NaCl-EDTA and PMSF 3 mmol/L was added to plasma, and another centrifugation (13,000 *g *for 90 minutes) was performed. The pellet was then resuspended in 100 μL of the same buffer. Under FORMVAR^® ^grids (Structure Probe, Inc West Chester, PA, USA), 10 μL of exosome samples was deposited for 30 seconds for film absorption. Thereafter, 1% phosphotungstic acid (10 μL) was added to the grids, and these samples were submitted to transmission electron microscopy in a Philips EM-420 microscope (Philips, Eindhoven, The Netherlands).

### Isolated heart preparation

Fifty male New Zealand rabbits weighing 2.5 to 3.5 kg were anesthetized with chloralose (60 mg/kg) and urethane (600 mg/kg) and were heparinized (250 IU). We performed a tracheotomy and submitted the rabbits to mechanical ventilation throughout the entire procedure. A torachotomy was performed, and the aorta was cannulated and perfused at a constant flow of 15 mL/minute with Krebs-Henseleit solution (composition NaCl 115 mM, KCl 5.4 mM, CaCl_2 _1.25 mM, NaHCO_3 _25 mM, MgSO_4 _1.2 mM, NaH_2_PO_4 _1.15 mM, and glucose 11 nM) at pH 7.40 equilibrated with 95% O_2_/5% CO_2 _and at a constant temperature of 37°C, in order to maintain the heart with a retrograde coronary perfusion, according to the Langendorff technique. The heart was excised, an incision was made in the left atrium, and a ventricular drain as well as a fluid-filled latex balloon were inserted through the mitral orifice into the left ventricle. The balloon was filled to achieve a constant diastolic pressure of 5 mm Hg. We recorded left ventricular pressure, and the maximal positive (+dP/dt_max_) and negative (-dP/dt_max_) left ventricular pressure derivatives were electronically derived from the left ventricular signal by means of a data acquisition system (Biopac MP100; Biopac Systems, Inc., Goleta, CA, USA). Hearts were maintained at 37°C throughout the experiment by enclosing the heart in a temperature-regulated double-glass chamber.

After a 15-minute stabilization period, we started the infusion of exosomes in a closed recirculating system (total volume of 100 mL) to achieve a concentration equal to one half of plasma concentration. This concentration was chosen after preliminary experiments depicted similar effects of exosome infusion in concentrations varying from 0.5 to 1× plasma concentrations. The system was maintained under perfusion for 20 minutes. After this period, the system was again perfused for 15 minutes with Krebs-Henseleit solution free of exosomes. In another set of experiments, the system was perfused 20 minutes before and during exosomes infusion with specific reactive oxygen species (ROS) inhibitors (Table 1) in order to confirm or disclose involvement of different ROS generation pathways.

**Table 1:** Targeted reactive oxygen species generation pathways

Inhibitor (final concentration)	Pathway inhibited
Diphenylene iodonium (0.02 mM)	Flavoenzymes, in particular NADPH oxidase
Indomethacin (0.1 mM)	Cyclooxygenase
Apocynin (1 mM)	NADPH oxidase
*N*-acetyl cysteine (3 mM)	Anti-oxidant; thiol group donor
L-monomethyl-arginine (0.1 mM)	Nitric oxide synthase

To investigate the effects of exosome infusion in an experimental model more closely related to human septic shock, rabbits were challenged with endotoxin (lipopolysaccharide [LPS] *Escherichia coli *serotype 026:B6) in a concentration of 1 mg/kg 6 hours before sacrifice. Three hours after exposure, rabbits were resuscitated with 8 mL/kg saline to reduce hypovolemia, which is a hallmark of this model. After 6 hours of LPS exposure, the hearts were removed and the experiments were performed as already described.

### Isolated papillary muscle preparations

Ten Wistar male rats weighing 350 to 450 g were anesthetized with ketamine and xylazine (0.1 mL per 100 mg of body weight each). A torachotomy was performed, and the heart was excised and immediately immersed in oxygenated (95% O_2 _and 5% CO_2_) Krebs-Henseleit solution (composition NaCl 118.5 mM, KCl 4.69 mM, CaCl_2 _2.52 mM, NaHCO_3 _25.88 mM, MgSO_4 _1.16 mM, KH_2_PO_4 _1.18 mM, and glucose 5.50 mM) in a bath at 29°C. The left ventricular chamber was opened, and anterior and posterior papillary muscles were dissected. Two stainless steel rings held the extremities of the muscles, and the muscles were vertically mounted in an organ bath filled with 35 mL of the same Krebs-Henseleit solution. The upper extremity of the muscle was connected by the ring to a force transducer (Grass FT 03 model; Grass Technologies, West Warwick, RI USA), and the inferior ring was connected to a micromanipulator (Mitutoyo model 2046F; Mitutoyo Corporation, Aurora, IL, USA). The muscles were stimulated electrically at 0.2 Hz and at a voltage approximately 10% above threshold by rectangular pulses of 5-millisecond duration through two longitudinally placed platinum electrodes. The preparation was stabilized for 30 minutes at the muscle length at which the maximal active tension was developed. After stabilization, exosomes from septic patients and healthy volunteers in a concentration equal to one half of plasma concentration were incubated in bathing solution and maintained for 45 minutes. The parameters recorded were developed tension (DT), resting tension (RT), positive temporal derivative of developed tension (+dT/dt), and negative temporal derivative of developed tension (-dT/dt). These two derivatives report temporal variations in contraction and relaxation myocardial capabilities, respectively.

### Organ chamber experiments

Rat thoracic aortas from 10 Wistar rats were carefully dissected and divided in four 5-mm-long rings that were suspended in two intraluminal parallel wires, introduced in an organ bath containing Krebs-Hepes at a constant temperature of 37°C, and equilibrated with 95% O_2_/5% CO_2_. An initial isometric contraction period with a DT of 2 g for 60 minutes was performed. During this period, two segments were incubated for 2 hours with exosomes from septic patients and from healthy volunteers in a concentration equal to plasma concentration. Immediately before the phase of preconstriction, a third ring was incubated with the same concentration of exosomes from septic patients. All the vessels were precontracted with norepinephrine 10^-7 ^M, and vascular tension was registered every 2 minutes. After a period of 15 minutes for equilibrium, concentration response curves to acetylcholine (10^-9 ^to 3 × 10^-5 ^M) were performed. In other experiments, vascular rings were incubated with exosomes overnight and analyzed the next day as described above.

### 4,5-Diaminofluorescein-derived fluorescence in exosomes

Recently, observations from the laboratory of one of the authors (MJ) demonstrated the presence of inducible NO synthase (NOS) in exosomes from septic shock patients [16]. Thus, we carried out these experiments in order to assess NO production from septic exosomes by using the fluorescent probe 4,5-diaminofluorescein (DAF)-2 (Calbiochem, now part of EMD Biosciences, Inc., San Diego, CA, USA). This method is based on the reaction of DAF-2 with NO in the presence of O_2 _under neutral pH, yielding the highly fluorescent DAF-2T. DAF-2 (5 μM) was added to a suspension containing 10 μg of exosome protein in 100 μL of Krebs buffer (pH 7.4). After a stabilization period of 5 minutes, fluorescence measurements were acquired at 37°C in an Amersham FARCyte/Tecan Ultra fluorescence plate reader (Amersham Biotech, now part of GE Healthcare, Little Chalfont, Buckinghamshire, UK). Excitation wavelength was set to 495 nm, emission was set to 520 nm, and measurements corresponding to 40-microsecond integration of signals were obtained by 10 flashes. In some experiments when indicated, N(G)-nitro-L-arginine methyl ester (L-NAME) (100 μM) or superoxide dismutase (SOD) (250 IU/mL) was added to the exosomes 20 minutes before DAF-2. Autofluorescence was corrected for by the inclusion of parallel blanks and did not exceed 10% of the total fluorescence. Data are expressed as arbitrary fluorescence units per milligram of exosome protein.

### Nitrate content in septic exosomes and in myocardial tissue

Samples of exosomes from septic patients were separated from their protein content by diluting the samples in an equal volume of trichloroacetic acid (10%) followed by centrifugation at 10,000 rpm for 10 minutes. Their intrinsic nitrate content was measured by chemiluminescence in a Sievers analyzer (model 280; Sievers Instruments, Inc., Boulder, CO, USA) with VCl_3 _and HCl (at 95°C) as reductants. Results were normalized for protein concentration.

In other experiments, isolated rabbit heart preparations were exposed for 45 minutes to exosomes of septic patients and healthy volunteers. After exposure, the system was perfused for 5 minutes with Krebs-Henseleit solution at 4°C free of exosomes. The heart was removed, and myocardial tissue was carefully dissected and immersed in liquid nitrogen. The hearts were minced under liquid nitrogen, and the homogenate was resuspended in 1 mL of Tris HCl buffer (50 mM, pH 7.40) containing mercaptoethanol (0.1%) and PMSF (1 mM) and centrifuged at 5,000 *g *for 5 minutes at 4°C. The supernatant was collected, and protein was quantified by the Bradford method. Samples (20 μL) had their nitrate content measured as described before, and the results were normalized for myocardial protein concentration.

### Statistical analysis

Data were considered normal using the Kolmogorov-Smirnov goodness-of-fit model and are presented as mean ± standard error of the mean. Single means were compared with the Student *t *test and paired *t *test as indicated, and a *p *value of less than 0.05 was considered significant. Means within group and between groups as well as the factor × time interaction were analyzed using two-way analysis of variance (analysis of variance two-way) with Bonferroni's correction for multiple comparisons. *Post hoc *analysis was performed with the Tukey test. The software used was Sigma Stat 2.0 software (Systat Software, Inc., San Jose, CA, USA).

## Results

### Electron micrograph of exosomes

**Figure 1:**
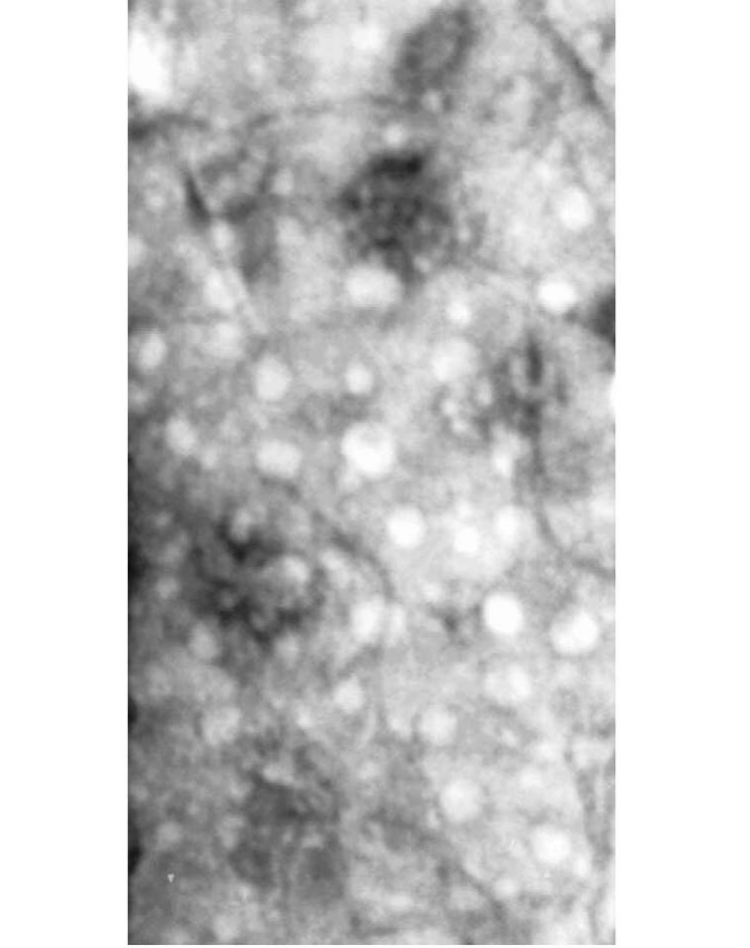
Exosomes from patients with septic shock. Electron micrograph of exosomes isolated from the plasma of patients with sepsis, showing round particles with diameters ranging from 50 to 150 nm. Magnification, × 41,000.

Figure 1 depicts transmission electron micrography of exosomes from septic patients. Exosomes were identified as round particles with diameters ranging from 50 to 150 nm, consistent with previous reports [13], with no deposit of electron-dense material (negative stain). Some larger particles can also be seen in the preparation, probably corresponding to microparticles derived from plasma membranes.

### Baseline heart function data

Table 2 depicts baseline data from isolated heart and isolated papillary muscle preparations. There were no significant differences in these parameters before infusion of exosomes from septic patients or healthy volunteers.

**Table 2:** Baseline data from isolated heart and isolated papillary muscle preparations

	Isolated heart			Isolated papillary muscle			
Parameters	Heart rate (bpm)	+dP/dt_max _(mm Hg/s)	-dP/dt_max _(mm Hg/s)	DT_max _(g)	Resting tension (g)	+dT/dt (g/s)	-dT/dt (g/s)
Septic exosomes	123 ± 4	3,676 ± 366	2,925 ± 336	5.3 ± 0.4	0.75 ± 0.07	52.6 ± 5.5	22 ± 3
Control exosomes	128 ± 3	3,136 ± 340	2,321 ± 469	5.7 ± 0.4	0.74 ± 0.1	63.8 ± 4.5	32.7 ± 2
*P *value	0.562	0.352	0.286	0.545	0.936	0.218	0.05

Data for isolated heart preparations are from nine experiments for septic exosomes and five experiments for control exosomes; data for isolated papillary muscle preparations are from eight experiments for septic exosomes and four experiments for control exosomes. +dP/dt_max_, maximal positive derivative of left ventricular pressure; -dP/dt_max_, maximal negative derivative of left ventricular pressure; DT_max_, maximal developed tension of isolated papillary muscle; +dT/dt, positive temporal derivative of developed tension; -dT/dt, negative temporal derivative of developed tension.

### Myocardial depressant effects of exosomes

**Figure 2:**
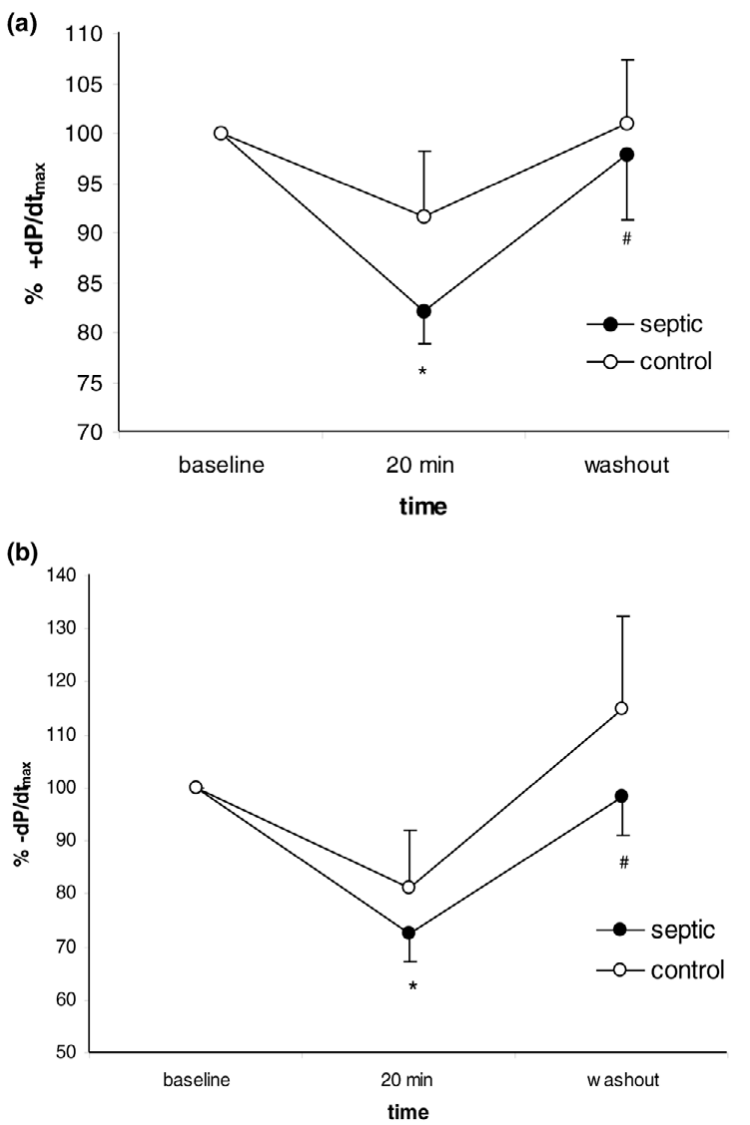
Effect of exosomes on left ventricular pressure in isolated rabbit heart preparations. Time course of positive **(a) **and negative **(b) **derivatives of left ventricular pressure after infusion of exosomes from septic patients and healthy volunteers. Data are mean ± standard error of the mean of nine experiments for exosomes derived from septic patients and five experiments for exosomes from healthy individuals. *p < 0.05 versus baseline; ^#^*p *< 0.05 versus 20 minutes (analysis of variance two-way, Tukey test). +dP/dt_max_, maximal positive derivative of left ventricular pressure; -dP/dt_max_, maximal negative derivative of left ventricular pressure.

Figure 2a,b depicts the effects of exosome infusion on positive and negative dP/dt_max _in isolated heart preparations. Incubation of the recirculating system with exosomes derived from septic patients at 0.5× plasma concentration induced a statistically significant decrease in myocardial contractility assessed through both positive and negative derivatives of left ventricular pressure when compared with baseline. This effect, however, was not statistically significant when compared with control exosomes. After 20 minutes, the exosome preparation was removed from the system and there was a spontaneous return of myocardial function to baseline levels after 15 minutes, indicating that the effects are reversible. Incubation with exosomes obtained from healthy individuals induced a non-significant decrease in myocardial contractility (*p *values at 20 minutes were 0.536 for +dP/dt_max _and 0.306 for -dP/dt_max_).

### Effects of reactive oxygen species inhibitors in exosome-induced myocardial dysfunction

**Figure 3:**
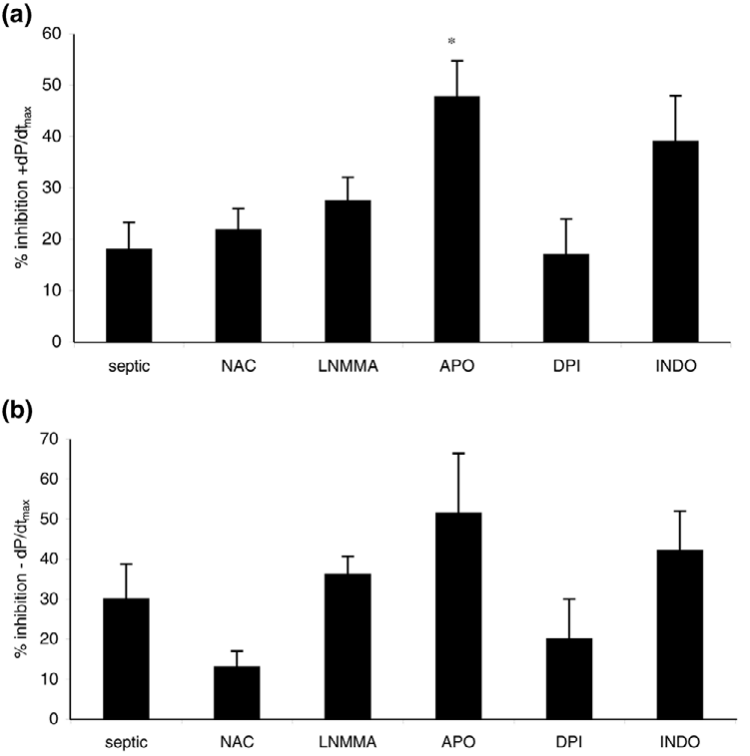
Effect of reactive oxygen species (ROS) inhibitors on exosome-induced inhibition of left ventricular pressure. Effect of ROS inhibitors on exosome-induced inhibition of maximal positive **(a) **and negative **(b) **derivatives of ventricular pressure. Data are mean ± standard error of the mean of nine experiments for exosomes from septic patients, four experiments for septic exosomes + L-monomethyl-arginine (LNMMA), five experiments for septic exosomes + n-acetylcysteine (NAC), four experiments for septic exosomes + dyphenyleneiodonium (DPI), three experiments for septic exosomes + apocynin (APO), and three experiments for septic exosomes + indomethacin (INDO). *p < 0.05 versus septic; (analysis of variance two-way, Tukey test). +dP/dt_max_, maximal positive derivative of left ventricular pressure; -dP/dt_max_, maximal negative derivative of left ventricular pressure.

To identify the mechanisms inducing exosome-dependent myocardial dysfunction, we performed experiments in which the perfusate was preincubated for 20 minutes with ROS inhibitors or redox-active compounds (Table 1) before exosome exposure. Overall, the reduction in positive and negative derivatives of ventricular pressure induced by exosomes and demonstrated in Figure 2 was largely not affected by the inhibitors tested (Figure 3a,b). Of note, however, the NAD(P)H oxidase antagonist apocynin induced a larger and statistically significant decrease in positive dP/dt_max_, an effect not dependent on a direct action of the inhibitor on myocardial performance, since preincubation of the heart with apocynin before exosome exposure did not induce myocardial dysfunction (data not shown). We postulated that this effect could be mediated by increased availability of NO, due to apocynin-mediated decrease in superoxide production. Nevertheless, an apparent obstacle to this proposal was that NOS inhibition did not correct the loss in myocardial contractility. This result, however, should be taken with caution since NOS inhibition can promote significant vasoconstriction and lead to myocardial dysfunction [17].

### Effect of exosome infusion in endotoxemic hearts

**Figure 4:**
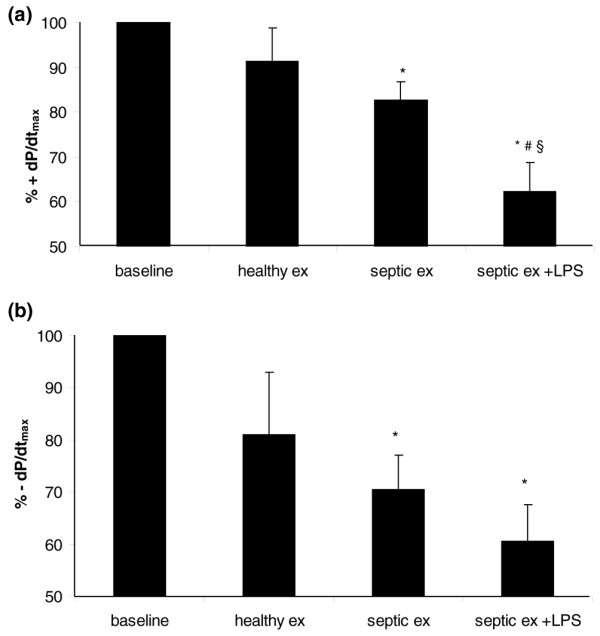
Amplification of the effects of exosomes on left ventricular contractility upon previous exposure to lipopolysaccharide (LPS). Effect of exosomes on percentage of maximal positive **(a) **and negative **(b) **derivatives of left ventricular pressure in endotoxemic hearts. Data are mean ± standard error of the mean of experiments with exosomes from septic patients on normal hearts (septic ex, *n *= 9), exosomes from healthy individuals on normal hearts (healthy ex, *n *= 5), and exosomes from septic patients in endotoxemic hearts (septic ex + LPS, *n *= 4). *p < 0.05 versus baseline; ^#^*p *< 0.05 versus septic ex; ^§^*p *< 0.05 versus healthy ex (analysis of variance two-way, Tukey test). +dP/dt_max_, maximal positive derivative of left ventricular pressure; -dP/dt_max_, maximal negative derivative of left ventricular pressure.

Hearts from endotoxemic rabbits were exposed to exosomes from septic individuals (Figure 4a,b). The mean baseline values obtained for positive and negative derivatives of ventricular pressure from endotoxemic hearts were, on average, 35% less than the values obtained from previously normal hearts (endotoxemic rabbits: +dP/dt_max_, 1,743.4 ± 266 mm Hg/s; -dP/dt_max_, 1,225.1 ± 231 mm Hg/s). Despite the baseline dysfunction, infusion of exosomes obtained from septic patients in endotoxemic hearts induced a significant decrease in contractility when compared with baseline and, in addition, a significant decrease in +dP/dt_max _as compared with infusion of septic and healthy exosomes in normal hearts not previously exposed to LPS. These results indicate that, in myocardial tissue primed by prior LPS exposure, exosomes may induce a more important negative inotropic effect.

### Isolated papillary muscle preparations

**Figure 5:**
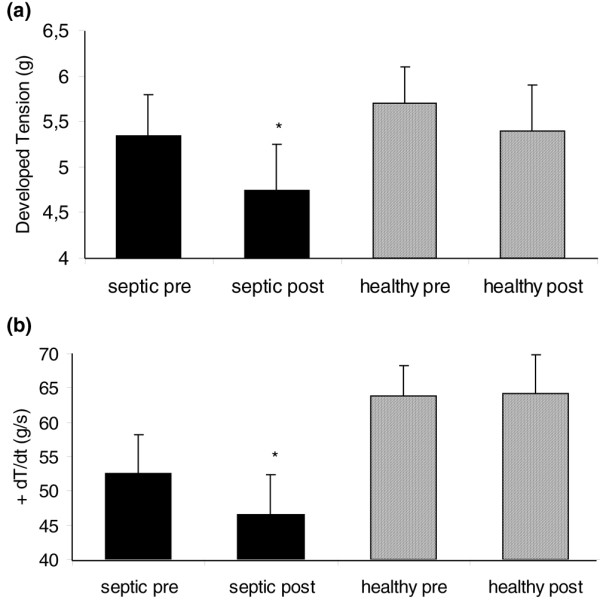
Effect of exosomes in isolated rat papillary muscle preparations. Effect of exosomes on developed tension **(a) **and its positive derivative (+dT/dt) **(b) **in isolated rat papillary muscle preparations. Data are mean ± standard error of the mean of eight experiments for papillary muscles before and after incubation with exosomes derived from septic patients (septic pre and post) and four experiments for papillary muscles before and after incubation with exosomes derived from healthy individuals (healthy pre and post). *p < 0.05 versus septic pre (paired *t *test).

Although isolated heart preparations are well-established systems for measuring myocardial performance, they may be subject to several pitfalls. To expand our results with isolated hearts, we incubated isolated rat papillary muscle preparations for 45 minutes with exosomes from septic patients and healthy volunteers at 0.5× plasma concentration. Exosomes from septic patients induced a statistically significant decrease in maximal DT and +dT/dt (Figure 5a,b), whereas exosomes from control individuals did not induce a decrease in these variables. Exosomes from septic patients or healthy volunteers did not induce a decrease in RT nor in its rate of temporal variation (data not shown). These results show a consistent inhibitory effect of the preparation of exosomes in myocardial function.

### Effect of exosomes in vascular reactivity

Standard curves of norepinephrine-mediated contraction and acetylcholine-mediated relaxation did not depict a significant direct effect of exosomes in vascular tone (data not shown). These data were assessed with concentration of exosomes equal to plasma concentrations and different periods of incubation (2 hours and overnight incubation).

### Nitric oxide production by exosomes and nitrate content in myocardium

**Figure 6:**
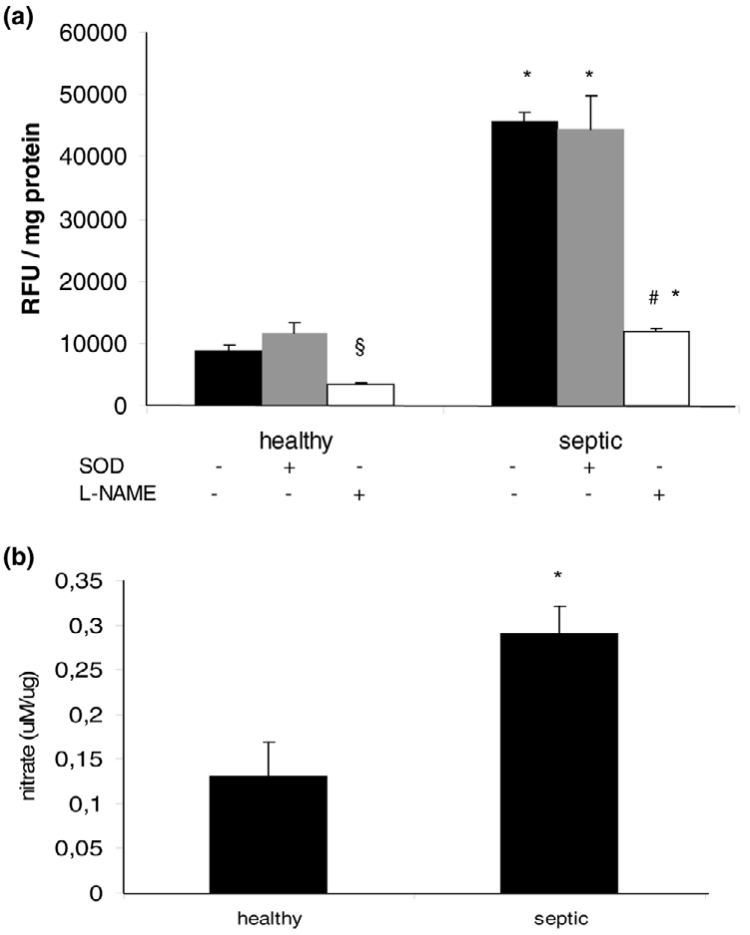
Exosomes exhibit intrinsic nitric oxide (NO) production and induce myocardial NO production. **(a) **4,5-diaminofluorescein-2 (DAF-2) fluorescence (an index of NO production) by exosomes from septic shock patients and healthy volunteers. Data are mean ± standard error of the mean (SEM) of six experiments with exosomes from healthy volunteers and septic patients. *p < 0.05 versus healthy; ^§^*p *< 0.05 versus baseline healthy; ^#^*p *< 0.05 versus baseline septic (one-way analysis of variance, Student-Newman-Keuls test). Black bars: baseline septic and healthy exosomes; gray bars: septic and healthy exosomes + superoxide dismutase (SOD) (250 IU/mL); white bars: septic and healthy exosomes + N(G)-nitro-L-arginine methyl ester (L-NAME) (100 μM). **(b) **Myocardial nitrate content of hearts exposed for 45 minutes to exosomes from septic patients or healthy volunteers. Data are mean ± SEM of four experiments for exosomes of septic patients and healthy volunteers. *p < 0.05 versus healthy (*t *test). RFU, relative fluorescence unit.

Figure 6a depicts NO production assessed by DAF fluorescence from samples of exosomes from septic patients and healthy subjects. Exosomes from septic patients induced significantly increased production of NO when compared with those from healthy volunteers. Specificity of DAF assay was confirmed by the inhibitory effects of L-NAME and by the absence of effects of SOD.

Exosomes from septic shock patients had an intrinsic nitrate content of 0.026 ± 8.5 μM per microgram of exosome protein. The nitrate myocardium content of hearts incubated for 45 minutes with normal and septic exosomes is demonstrated in Figure 6b. As shown, septic exosomes induced a significant increase in myocardial nitrate content. Taken together, these results indicate an intrinsic production of NO by exosomes as well as an increase in the myocardial NO pool induced by exosomes from septic individuals. This activation may be linked to myocardial dysfunction.

## Discussion

In this study, we showed for the first time that (patho)physiologically relevant concentrations of platelet-derived exosomes from septic patients induce a decrease in myocardial contractility in isolated heart and papillary muscle preparations. The effect of exosomes is enhanced by previous exposure to LPS. Therefore, exosomes may be implicated in myocardial dysfunction of sepsis. Exosomes also contain NOS, generate NO, and induce myocardial NO production. These data provide evidence that NO may be a pathway for septic exosome-derived myocardial dysfunction.

In our Langendorff model, exosomes induced a significant decrease in myocardial contractility assessed by positive derivative of ventricular pressure. However, in these preparations, myocardial contractility is dependent on several variables, such as coronary perfusion pressure and heart rate, and compounds affecting these indices may not have an intrinsic myocardial depressant effect. Thus, we decided to perform some experiments in a system in which the effect of exosomes in myocardial contractility could be measured. The experiments with isolated papillary muscles demonstrated unequivocally an inhibitory effect of exosomes in myocardial function. However, in isolated papillary muscles, exosomes decreased myocardial performance only in the DT and its positive temporal derivative. This finding may be due to the possible dependence of myocardial relaxation on some peculiarities of the isolated heart, such as changes in transmural perfusion. The lack of a direct effect of exosomes in vascular tone evidenced in this study argues against a direct effect of exosomes in coronary tone. This result also contrasts with the decreased relaxation verified after exposure of vessels to other types of microparticles, such as vesicular particles exposing phosphatydilserine [10].

In LPS-treated rabbits, exosomes induced more significant decreases in myocardial function. It is noteworthy that these preparations had baseline levels of dP/dt, on average, 65% lower than normal hearts. The enhanced effect of exosomes in myocardial contractility of septic hearts may be partially explained by the results obtained by Clayton and colleagues [18] which suggest that, in systems previously activated by inflammatory mediators, exosomes may have their effect enhanced due to their increased adherence to target cells promoted by adhesion molecule exposure.

Although the experiments with inhibitors could not clarify the precise mechanism of myocardial dysfunction, they were important for ruling out several possible pathways. In particular, experiments with apocynin, a specific NADPH oxidase inhibitor, called our attention to the possibility of NO-mediated dysfunction. Our results with L-monomethyl-arginine do not rule out NO as the mediator of myocardial dysfunction, probably due to coronary vasoconstriction induced by NO antagonism [17].

NO has been implicated for a long time in sepsis-induced myocardial dysfunction. Classic studies have linked cytokine production to cardiac dysfunction mediated by myocardial NOS [19,20]. One study reported that, immediately after incubation of isolated papillary muscles with cytokines such as TNF-α and IL-6, there was a concentration-dependent and reversible inhibition of myocardial contractility [19], an effect very similar to our results. In that study, incubation with NO inhibitors blocked the depression, but removal of endocardial endothelium did not alter the response. The authors suggest that the enzyme probably involved in this dysfunction is constitutive NOS (NOS3) since the rapid onset of the effect did not suggest a mechanism requiring gene transcription [19].

Inducible NOS (NOS2) has also been linked to myocardial dysfunction of sepsis [21-23]. Mice lacking NOS2 are protected against endotoxin-induced myocardial depression, and inhibition of this enzyme with specific compounds prevents this effect [22]. Since our exosomes carry NOS2 and their incubation promoted increased myocardial content of NO, it is also possible that the presence of this enzyme may stimulate myocardial production of NO, thus contributing to our results.

Another possible source of myocardial depression is peroxynitrite and/or related toxic by-products of reaction between NO and superoxide. Ferdinandy and colleagues [7] demonstrated that infusion of cytokines such as TNF-α or IL-1-β is associated with impairment of cardiac mechanical function in isolated hearts, accompanied by increased myocardial activities of NOS2, xanthine oxidoreductase, and NADH oxidase. The levels of nitrotyrosine and dityrosine (markers of peroxynitrite formation) were enhanced in the perfusate, whereas treatment with ROS scavengers such as tiron and NOS inhibitors blocked myocardial dysfunction [7]. The authors concluded that ROS (particularly peroxynitrite) were involved in cytokine-induced myocardial dysfunction. However, peroxynitrite is not a good candidate to underlie our effects since treatment of the hearts with ROS inhibitors did not reverse the inhibitory effect of exosomes.

Although our results are encouraging, some study limitations should be considered. First, the isolated heart or papillary muscle preparations provide interaction of exosomes with different cell types. Thus, our results should not be taken to represent exclusively the effects of exosomes with cardiomyocytes. Particularly, in isolated hearts, effects of exosomes on coronary vascular tone could account for the observed dysfunction, a limitation overcome in the papillary muscle preparation and also discouraged by the absence of an effect of exosomes on isolated vessels. On the other hand, taken together, the strength and reproducibility of the effects in both preparations suggest at least some direct effect of exosomes on myocardial contractility. A puzzling question was the observed effect of healthy exosomes in the myocardium. Although this effect was small and not statistically significant versus baseline, it was sufficiently evident to jeopardize a statistical difference with septic exosomes. Indeed, there is evidence in the literature of a cellular effect of 'sham' exosomes, mainly related to ROS production [9,24]. Thus, it is apparent that the observed effect of septic plasma exosomes may be due both to an increased number of exosomes as well as an intrinsic property of such particles. Since a reliable method of exosomes quantitation in plasma is not available, this issue remains open. In any case, however, our data clearly show that myocardial effects of exosomes make a potentially important contribution to septic myocardial dysfunction.

## Conclusion

This study showed that (patho)physiologically relevant concentrations of exosomes derived from platelets of patients with septic shock may induce myocardial dysfunction in isolated rabbit hearts and isolated papillary muscle preparations. The increase in myocardial dysfunction after LPS exposure and the increase in myocardial content of NO derivatives as well as the production of NO by exosomes from septic patients suggest that NO may be a significant contributor to this phenomenon. Although the significance of this dysfunction *in vivo *is not yet established, the data depicted here indicate that exosomes present in the circulation may represent a novel redox-signaling microenvironment and contribute to myocardial dysfunction of sepsis.

## Key messages

• Previous data have demonstrated the presence of exosomes in the plasma of septic shock patients.

• Exosomes derived from the platelets of septic shock patients induce myocardial dysfunction in isolated heart and isolated papillary muscle preparations.

• Previous exposure of hearts to lipopolysaccharide increases exosome-derived myocardial dysfunction.

• Data supporting nitric oxide (NO) production by exosomes and the increase in myocardial NO content after exosome exposure suggest that NO may be associated with the induction of myocardial derangement in this setting.

• Exosomes may represent a novel vascular redox-active pathway in sepsis.

## Abbreviations

DAF = 4,5-diaminofluorescein; +dP/dt_max _= maximal positive derivative of left ventricular pressure; -dP/dt_max _= maximal negative derivative of left ventricular pressure; DT = developed tension; +dT/dt = positive temporal derivative of developed tension; EDTA = ethylenediaminetetraacetic acid; IL = interleukin; L-NAME = N(G)-nitro-L-arginine methyl ester; LPS = lipopolysaccharide; NO = nitric oxide; NOS = nitric oxide synthase; NOS2 = inducible nitric oxide synthase; PMSF = phenylmethylsulfonyl fluoride; ROS = reactive oxygen species; RT = resting tension; SOD = superoxide dismutase; TNF-α = tumor necrosis factor-alpha.

## Competing interests

The authors declare that they have no competing interests.

## Authors' contributions

LCPA conceived the study, carried out the experiments, analyzed and interpreted the data, and wrote the manuscript. MJ helped to conceive the study, carried out the experiments in ROS production, analyzed and interpreted the data, and corrected the final version of the manuscript. VP carried out the experiments with isolated heart preparations, analyzed the data, and helped to write the draft of the manuscript. MAP carried out the experiments, helped to write the draft of the manuscript, and corrected the final version. EB carried out organ chamber experiments and collected, analyzed, and interpreted the data. PJFT participated in the design of the study, carried out papillary muscle experiments, and analyzed and interpreted the data. FRML conceived and supervised the study, analyzed and interpreted the data, and corrected the final version of the manuscript. All authors read and approved the final manuscript.
